# The Growth Model of Forensically Important *Lucilia sericata* (Meigen) (Diptera: Calliphoridae) in South Korea

**DOI:** 10.3390/insects12040323

**Published:** 2021-04-06

**Authors:** Sang Eon Shin, Ji Hye Park, Su Jin Jeong, Seong Hwan Park

**Affiliations:** Department of Legal Medicine, Korea University College of Medicine, Seoul 02841, Korea; shinfbr@nate.com (S.E.S.); jhp09@korea.kr (J.H.P.); rainofsujin@naver.com (S.J.J.)

**Keywords:** development, minimum postmortem interval (PMI-min), rearing, calliphoridae, *Lucilia sericata*

## Abstract

**Simple Summary:**

This study provides a detailed growth data for Lucilia sericata (Meigen) collected in South Korea. With the growth data, authors compared different minimum ADH models and found little differences. However, the logalithmic model was the best fit among differenct models.

**Abstract:**

Development of forensically important *Lucilia sericata* (Meigen) was analyzed in South Korea. Rearing was replicated five times at seven constant temperatures between 20–35 °C to elucidate changes in accumulated degree hours, based on developmental stage and body length, and 2673 individuals were statistically analyzed. The results indicated that the optimum temperature, the base temperature, and the overall thermal constant were 22.31 °C (±1.21 °C, 95% CI), 9.07 °C, and 232.81 ± 23 (mean ± SD) accumulated degree days, respectively. In the minimum ADH models of each development stage, nonlinear regression graphs were parallel at the immature stages. Based on the scatter plot (*n* = 973) of immature stages using ADH values and body length, the logarithmic model using Log_10_ADH as the dependent variable was identified as the best fitting regression model. Additionally, the adjusted *R*^2^ value and mean square of error were 0.911 and 0.007, respectively. This is the first forensically focused study on the development of *L.* *sericata* for the estimation of minimum postmortem interval in South Korea. In future studies, we intend to study the development of other necrophagous fly species and to identify parameters for the determination of age at post-feeding and pupal stages.

## 1. Introduction

In medico-legal entomology, insects are used as scientific evidence to solve cases related to the time since death (TSD), entomotoxicology, abuse, and neglect, etc. This field focuses primarily on the time at which insect eggs (or larva in Sarcophagidae) are laid on the body after death to estimate the minimum postmortem interval (PMI-min) [1]. PMI-min is assumed to be most accurately predicted by calculating the age of immature insects [2], even when the body is badly decayed. As a real case, fly pupae in the soil and maggots found in the freezer for body preservation were collected 45 days after the discovery of a putrefied male cadaver in fallow ground. The PMI-min was estimated at 10 days before the discovery of the body, based on egg laying time from the growth rate of insects, the distribution of pupae toward pupariation sites, weather information, and so on [3].

The growth rate of insects is strongly influenced by temperature and can be presented as an S-shaped velocity curve at constant temperatures [4]. Further, the growth rate of immature insects is considered to have a linear relationship with developmental temperatures [5]. In these linear models, energy budgets designated for physiological development are considered to remain constant through the various developmental stages [5]. However, base temperature varies from species to species and can also vary with geographic location [5]. Considering these dependent relationships, the values of accumulated degree hours or days (ADH or ADD, respectively) for specific developmental stages (while estimating the age of forensically important insects) and the base temperature should be predetermined by setting constant temperatures in rearing experiments [5,6,7].

*Lucilia sericata* (Meigen), the earliest arriving necrophagous fly species on corpses, is known as one of the most dominant forensically important species in the temperate zone of the Northern Hemisphere as well as in both urban and suburban areas [2,7,8] and has been found in such places as apartments in Germany [9], some stagnant water in a city of Spain [10], the Iwate prefecture in Japan [11], indoors in Italy [12], and indoors in South Korea [13]. In addition, this species is causing myiasis in South Korea, and the importance of myiasis with this species as an indicator of a poor hygienic condition and a lack of due care is ever growing in an aging society such as is South Korea [14]. Consequently, numerous studies on the growth of *L. sericata* have been performed in several countries [15,16,17,18,19,20]. Anderson [18] documented the minimum and maximum time taken to reach each developmental stage as a way to estimate the time since death. Shortly afterward, Grassberger and Reiter [19] illustrated morphological length and stage changes using isomegalen- and isomorphen-diagrams. Nevertheless, Roe and Higley [21] emphasized that methodological inconsistencies in the previous studies made it difficult to apply error rates or confidence intervals to cases within a given region. These inconsistencies stimulated the launch of studies on blind validation [22] and field validation [23] of development datasets.

The goal of the present study was to generate practical development data for *L. sericata*, the most common indoor insect species in South Korea [13]. The rearing experiments were replicated five times at seven different temperatures, held constant throughout the investigation, to analyze the changes in ADH or ADD according to developmental stage and body length.

## 2. Materials and Methods

### 2.1. Identification and Rearing of Adult Flies

**Figure 1:**
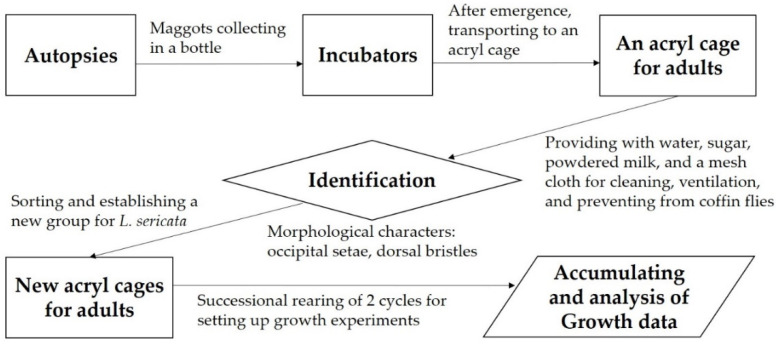
A diagram showing the preparation process for the growth experiment of *Lucilia sericata.*

Maggots of *L. sericata* were collected from autopsies in northeastern Seoul, Korea. After their emergence in incubators, the adults were identified by the following morphological characters: 6 to 8 occipital setae behind the vertical bristle, acrostichal bristles 2 + 3, and dorsocentral bristles 3 + 3 [24]. The adult flies were provided with a damp paper towel as a water source, along with a mixture of powdered milk (50%) and dry granular sugar (50%). Newly identified adult flies were occasionally added to acryl cages (the dimensions of 40 × 40 × 40 cm^3^), which were constructed to prevent odor and the trapping of flies in the folds. A mesh cloth (20 × 20 cm) was used for the lateral sides and was attached using Velcro tape. This design facilitated internal cleaning and also provided ventilation. Moreover, the size of the mesh was small enough to prevent the intrusion of coffin flies (Phoridae) (Figure 1).

### 2.2. Sampling and Rearing of Maggots

Fresh pork liver was sliced into pieces (approximately 50 g in weight) and the pieces were frozen at −20 °C until use. They were thawed slowly at 25 °C for 24 h in order to maintain freshness and minimize blood leakage. Eggs were collected from a piece of fresh pork liver within 40 min of the beginning of egg laying. Eggs were then separated from each other by soaking in sodium sulfate solution (2%) and rinsing with distilled water [19]. Twenty-five of these moist eggs were then deposited onto a new piece of fresh pork liver (50 g) using a small moist brush to prevent them from drying. Ten bottles (diameter 10 cm, height 9 cm) containing the eggs and liver were placed at the center of a growth chamber (50 × 50 × 50 cm) to reduce the effects of location. The process of rearing—from eggs to adult stages—was duplicated five times at 70% relative humidity with a photoperiod (h) of 16:8 (L:D) at seven constant temperatures—namely, 2 °C, 22 °C, 24 °C, 26 °C, 28 °C, 32 °C, and 35 °C (for a total of 35 experiments). This was done considering the possibility of diapause [18,25] and the upper temperature threshold [19,26]. When the movement trace of post-feeding larvae could be observed, dry wood sod (depth 6 cm) was added to the 10 bottles for pupation. Once the first adult fly emerged, the bottles were transferred to acryl cages to continue the rearing of adult flies. Notably, the selected temperature of the growth chambers was not assigned to a single temperature. This was done to distinguish between the effect of the selected temperature and the mechanical error of the growth chamber [27]. Moreover, the center temperature of the growth chambers was measured for temperature correction. Regardless of body length or developmental stage, four individuals were removed from one bottle every 12 h. Afterward, the same bottle was replaced, and the other bottles in the chamber were shuffled. Specimens (four individuals) were killed by submersion in boiling water for 30 s to prevent shrinkage. Specimens were then preserved in an 80% ethanol solution [28] and placed in a freezer (−20 °C).

### 2.3. Body Length, Larval Stages, and the Optimum Development Temperature

Body length was measured using micrographs (Olympus, SZX10) and calculated using Microsoft Office Excel 2007 (Microsoft Corp., Redmond, WA, USA) (Table S1). Larval stages were determined based on the condition of the crop and the number of posterior spiracle slits [5]. Additionally, the minimum time taken to reach each developmental stage was based on the time at which the first observed individual was discovered (Table S2). The optimum development temperature was statistically estimated from the inflection in the sigmoid model of growth rate [27]. Additionally, ADH (or ADD) was calculated using the following equations [5] (Table S3):

Time (h) × (temperature − base temperature) = ADH (°H)(1)

Time (days) × (temperature − base temperature) = ADD (°D)(2)

### 2.4. Data Fit and Statistical Analysis

SigmaPlot (version 10.0) and Microsoft Office Excel 2007 were used for plotting all graphs and for performing basic statistical analyses. Two-way ANOVA, without replication, was conducted using the SAS program (ProcMIXED, SAS9.4) [29] to determine the differences among minimum mean hours spent in each development stage and temperature (*p* ≤ 0.05) [23]. In addition, the growth data from egg to adult were fitted with a four-parameter sigmoid model to determine the minimum growth rate ($y^{0}$) as well as the optimum development temperature ($x^{0}$):

(3) $$F(x)\;=\;y^{0}\;+\;a/(1\;+\;\exp\;(-(x\;-\;x^{0})/b))$$

where $y^{0}$ is the minimum developmental rate, $x^{0}$ is the inflection or the optimum development temperature in the sigmoid curve, “a” is the difference between the maximum and minimum developmental rates, and “1/b” is the steepness of the sigmoid curve [27,30]. The fitted curve for growth rate was compared with rearing results from previous studies using 95% confidence and 95% prediction intervals. In addition, a scatter plot was produced to illustrate the correlations among the following variables: body length, ADH, and growth stage, including the transition stages in the first and second instar. Using the scatter plot, linear regression and nonlinear regression analyses were performed to conform to the growth model of the immature stages using ADH and Log_10_ADH values.

## 3. Results

### 3.1. Body Length and Minimum Development Time

**Figure 2:**
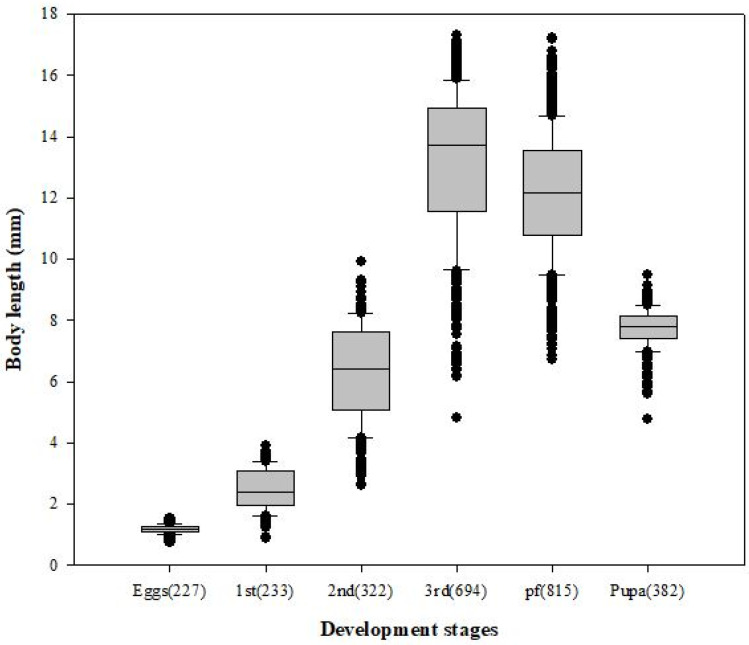
Boxplot of body length according to developmental stages in *L*. *sericata* (*n* = sample size). Body length was greatest at the feeding third instar stage but decreased during pupation.

Among 8750 eggs (25 eggs × 10 bottles × 7 temperatures × 5 replicates), 2673 individuals were sampled and statistically analyzed (sample coverage, 32.7%), including 200 outliers. Body length values (mean ± SD) were 1.17 ± 0.13 mm (egg), 2.45 ± 0.65 mm (first instar), 6.29 ± 1.54 mm (second instar), 13.16 ± 2.40 mm (third instar), 12.12 ± 1.99 mm (post-feeding larva), and 7.73 ± 0.63 mm (pupa) (Figure 2). Additionally, the minimum development time (mean ± SD, *n* = sample size) from egg to adult stages at each of the seven temperatures was 20.60 ± 1.53 days (20 °C, *n* = 499); 16.42 ± 1.54 days (22 °C, *n* = 357); 14.78 ± 0.61 days (24 °C, *n* = 361); 12.75 ± 0.96 days (26 °C, *n* = 332); 11.70 ± 0.84 days (28 °C, *n* = 360); 10.90 ± 0.55 days (32 °C, *n* = 390); and 10.70 ± 0.45 days (35 °C, *n* = 374). Values for the minimum mean development time were significantly different among the developmental stages (*F* = 53.8; df = 5; *p* ≤ 0.05) and temperatures (*F* = 3.6; df = 6; *p* ≤ 0.05).

### 3.2. Base Temperature, Optimum Temperature, and Comparisons with Previous Studies

**Figure 3:**
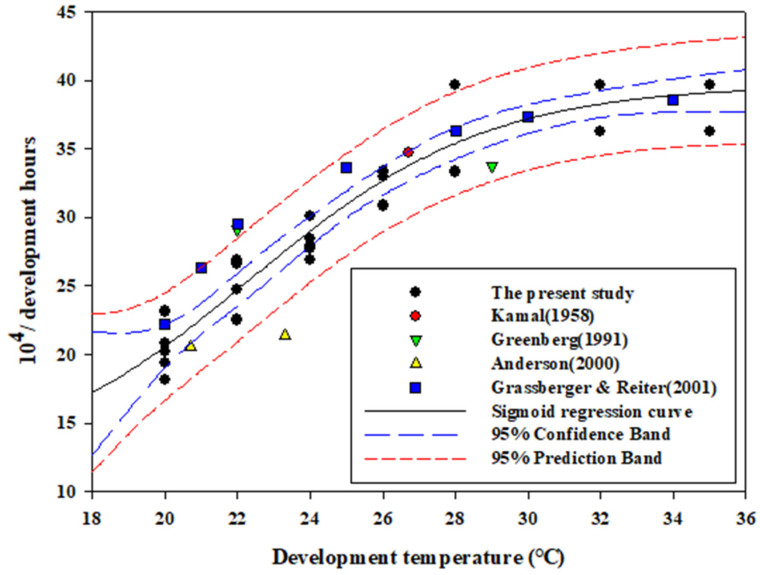
Four-parameter sigmoid model for the growth rate of *Lucilia sericata* from eggs to adults at five temperature regimes with 95% confidence and 95% prediction intervals. Although rearing was duplicated five times at seven temperatures, the dots of the present study overlapped due to similar results. Data from most previous studies fell within the 95% prediction interval.

The base temperature was calculated as 9.07 °C (Table 1), and the growth data were fitted with the four-parameter sigmoid model. The statistically adjusted *R*^2^ value was 0.93, and the mean square error (MSE) was 3.10; coefficient values were calculated as 28.56, 3.28, 22.31, and 11.14 for a, b, $x^{0}$, and $y^{0}$, respectively. The optimum temperature (or the inflection ($x^{0}$)) was estimated as 22.31 °C (±1.21 °C, 95% CI) (Figure 3). Additionally, the growth rate in the present study corresponded to that reported in most previous studies in the 95% prediction interval (Figure 3).

### 3.3. Minimum ADH Models and Scatter Plots

**Figure 4:**
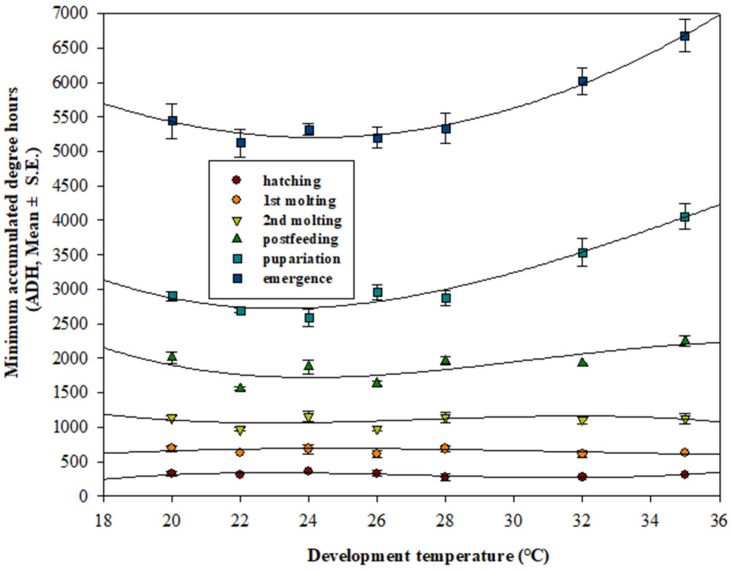
Minimum accumulated degree hours (ADH) model for *Lucilia sericata* developed by accumulated minimum development hours at each developmental stage, at seven temperature regimes, with a base temperature of 9.07 °C. Regression curves were fitted with polynomial cubic equations.

**Figure 5:**
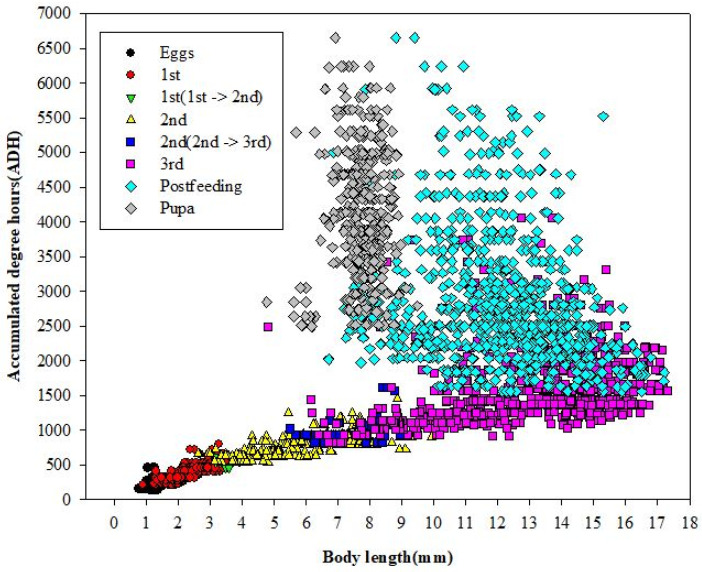
Scatter plot (*n* = 2566) of *Lucilia sericata* developed based on ADH values and body lengths at each developmental stage. It shows a constant relationship during feeding larval stages (≤1551.60 ADH), minimum ADH values for each developmental stage, and the possibility of transition forms as a forensic indicator.

When plotted, the minimum ADH models based on the same development stages ran parallel at feeding larval stages. However, the plots curved upward at the post-feeding and pupal stages (Figure 4). In addition, the scatter plots (*n* = 2566) developed from ADH values and body length presented a constant relationship during the feeding larval stage (≤1551.60 ADH). Moreover, minimum ADH values at each developmental stage were estimated as follows: first instar: 203.16 ADH; second instar: 524.64 ADH; third instar: 812.64 ADH; post-feeding third instar: 1551.60 ADH; and pupa: 2492.04 ADH (Figure 5). The first and second instar larvae transitioning to the next developmental stage and characterized by one additional slit under the posterior spiracle slits [4] presented relatively narrow ADH ranges—from 454.3 ADH to 622.3 ADH and from 812.6 ADH to 1612.4 ADH (Figure 5).

### 3.4. Linear and Nonlinear Regressions during Immature Stages

**Figure 6:**
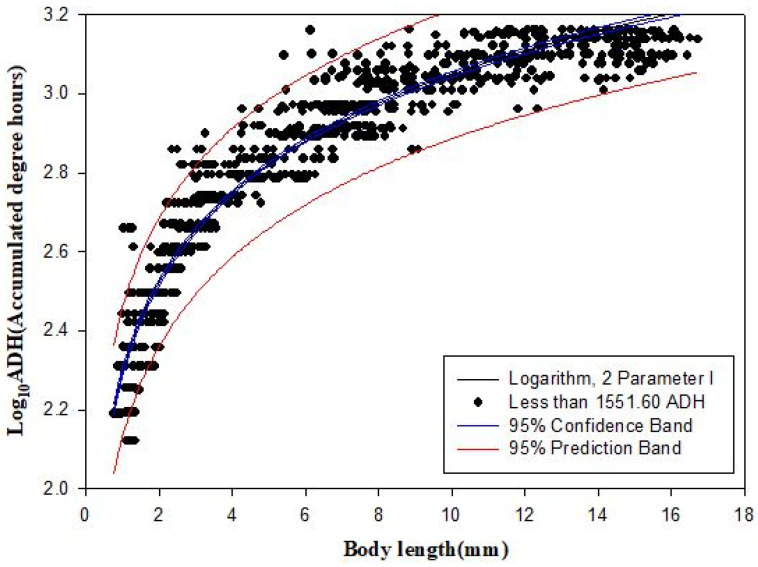
A nonlinear regression analysis was performed from the scatter plot (*n* = 973) of feeding larval stages (≤1551.60 ADH) of *Lucilia sericata* to determine the correlation between ADH values and body lengths. The adjusted *R*^2^ value and mean square error were 0.911 and 0.007, respectively.

Both linear regression and nonlinear regression were performed using ADH values (f(*x*)) and body length (*x*) during the feeding larval stage (≤1551.60 ADH) in the scatter plot (*n* = 973). It is important to note that the adjusted *R*^2^ value of the secondary model was higher than that of the linear model, whereas the MSE using Log_10_ADH was lower (Table 2). Therefore, the logarithmic model (2 Parameter I) using Log_10_ADH was estimated as the best fitting regression model (Figure 6), considering the *R*^2^_adj_ value (0.911) and MSE (0.007) (Table 2).

## 4. Discussion

The growth rate of insects is strongly influenced by temperature and is presented as an S-shaped velocity curve at constant temperatures [4]. In the present study, the forensically important *L. sericata* was reared under conditions that aligned with five criteria for controlling variation factors—namely, a food source of fresh pork liver thawed within 24 h [31], a photoperiod of 16 h (light) [25], the placement of rearing bottles in the center of the chamber with a thermometer [32], the number of eggs being limited to 25 to prevent heat generation by friction [33], and the random use of chambers to distinguish the effect of programed temperatures and mechanical errors [32].

In our pilot study, the sampling of entire-age cohorts at 20 °C to produce insect growth models [34] led to the number of bottles exceeding the capacity of a rearing chamber. This problem eventually caused poor ventilation, thereby reducing the effect of the programed temperature. Meanwhile, in the present study, four individuals were sampled from one of the ten shuffled bottles every 12 h, and rearing experiments were repeated five times at seven temperatures to meet the minimum sample size for statistical significance (*n* = 318) [35]. This was done in accordance with the sampling method outlined by Anderson [18]. In addition, because it is difficult to count moving first instar immediately after hatching, the hatch rate for 25 eggs could be estimated through the sum of the number of sampled individuals from egg to adult and the number of left puparia.

It is important to note that the minimum amount of time taken to reach each stage of development was not based on ecologically meaningful 50% transition times but rather on the observed time of the first individual [17,18,19]. This is because, in forensic science, the existence of each development stage in a scene becomes scientific evidence, and the best standard practice recommends collecting at least 10% of the total population to ensure the collection of the oldest (or the largest) insects [36]. In addition, it was confirmed that the previous growth data were mostly included within 95% prediction intervals based on the growth data of this study (Figure 2), despite differences in geographic region and type and properties of food. These results suggest that the application of a consistent analysis method of developmental stages, based on the observed time of the first individual (minimum), is more important than geographic region or food in insect growth model studies.

Currently, forensic entomologists need to know the minimum growth time of the oldest insect collected at a scene and also need to require information on the optimal temperature for laboratory rearing after sampling [27]. In a study of larval mass effect on *Lucilia sericata*, ambient temperatures between 22 °C and 25 °C were reported as the optimal temperature range for the highest heat emission per larva [37]. These results were consistent with the fact that 22.31 °C (±1.21 °C, 95% CI) was measured as the optimum temperature for this study, and it was thought to be related to the fact that the growth of insects is dependent on the temperature. Kotzé et al. (2015) found that the body length of *Lucilia cuprina* was greatest near the optimal temperature [38], and this result was also the same as our result, as mean body lengths of third instar were greatest at 24 °C (14.06 ± 1.85 mm).

For the estimation of base temperature from egg to adult, five temperatures between 20 and 28 °C were used on a linear growth graph, and minimum ADH values were determined using the x-intercept approach at 9.07 °C [39] (Table 1). Notably, this value is similar to the 9.0 °C reported by Marchenko [20]. This similarity suggests that there is little difference by geographical region between Russia and South Korea for *Lucilia sericata* and that methodological factors (food, larval mass effects, etc.) were consistent with this study, including the setting of the temperature range centering on the flection point of the sigmoid growth curve (the optimum temperature) [27].

The minimum ADH model of this study (Figure 4) was produced using the minimum time required to reach each developmental stage, from the initial egg laying phase, for easy identification of minimum ADH values in a scene. Reibe et al. (2010) [40] also published a ADH model similar to this study, but this study used a base temperature of 9.07 °C and growth data made of 25 eggs, whereas they used 8.0 °C and growth data made of 100 eggs by Grassberger & Reiter (2001) [19]. Unlike this study, according to Marchenko (1985) [41], their growth data were estimated to reflect the larval mass effects. Therefore, in order to reduce the estimation error of PMI-min, it was considered essential to select an appropriate ADH model according to the field situation even if the same species of insects were found.

Additionally, the regression curves in Figure 3 demonstrate the delay in pupariation due to the extension of the post-feeding larval stage, which resulted from the high programed temperature rather than the crowding of larvae [17]. We excluded any heat generated from the larval population because we had placed only few individuals in each bottle to avoid the heat generated by their bodies [33]. In addition, food and dry sawdust were sufficiently provided [42].

A practical ADH model should include useful parameters such as body length and growth stage, as well as prediction intervals. However, the isomegalen- and isomorphen-diagrams by Grassberger and Reiter [19] have no error values, and the ADH model by Reibe et al. [40] has no data for body length. For these reasons, a new scatter plot was designed to show body length, growth stage, and ADH values (Figure 5). The following is a summary of our findings. First, ADH values and body length during feeding larval stages have a linear relationship. Second, other parameters such as gene expression differences are needed for age prediction during the post-feeding third larval stage and the pupa stage [43]. Third, the minimum ADH values taken to reach each developmental stage can be determined from 20 to 35 °C, and lastly, the first and second instar larvae transitioning to the next developmental stage have potential as forensic indicators due to their relatively narrow ADH ranges.

In the feeding larval stages of the scatter plot, linear and nonlinear regressions were performed to understand the correlation between ADH values and body length. The best fit regression model was the logarithmic model (2 Parameter I) using Log_10_ADH as the dependent variable (Table 2), considering the *R*^2^_adj_ value and MSE. It was expected that using ADD, rather than ADH, as the dependent variable would result in a low MSE [44]. However, it was excluded from this study because it was thought that the precision was low, even though the measurement of the growth period in units of days had high accuracy. In addition, 95% of the prediction intervals in fitted models, or errors of ADH estimates from body length values, were due to variability within a species [27] and the sampling interval of 12 h. Therefore, shorter sampling intervals were suggested within a growth period of 120 h for a more precise estimation of PMI-min [1,34].

## 5. Conclusions

The growth models for Korean *L. sericata* showed little difference in this study when compared with the results of previous studies; minimum ADH values at each stage of development could be determined. Based on the scatter plot of ADH values and body length values at immature stages, the logarithmic model was the best fit. In addition, minimum ADH values and 95% prediction intervals at each body length value could be statistically estimated (adjusted *R*^2^ = 0.92). In future studies, it is our intention to rear subdominant necrophagous fly species and develop additional markers for age prediction at post-feeding and pupal stages.

## Figures and Tables

**Table 1:** Base temperatures and *p* values for *Lucilia sericata* according to development stage.

Stage	Regression Equation	*R* ^2^	Base Temperature (°C)	*p*-Value
Egg	Y = 39.9305x − 479.1665	0.8701	12.0000	0.0207 *
First instar	Y = 25.4630x − 164.0213	0.5673	6.4416	0.1416
Second instar	Y = 21.3656x − 147.9693	0.6338	6.9256	0.1071
Third instar	Y = 12.5778x − 92.7091	0.6265	7.3709	0.1106
Post-feeding	Y = 9.2416x − 65.3248	0.3773	7.0686	0.2703
Pupa	Y = 4.4917x − 46.8452	0.9152	10.4293	0.0108 *
Egg to adult	Y = 1.9040x − 17.2758	0.9907	9.0734	<0.0004 *

* *p*-value < 0.05.

**Table 2:** Linear and nonlinear regression models during immature stages using ADH and Log_10_ADH values (*n* = 973).

Model	Y	Regression Equation	*R* ^2^_adj_	SE	MSE
Linear	ADH	Y = 238.508 + 83.076x	0.872	142.800	204 × 10^2^
Quadratic	ADH	Y = 41.570 + 158.911x − 4.814x^2^	0.920	112.729	127 × 10^2^
Logarithm	ADH	Y = 38.392 + 466.960 Log_10_ (x)	0.899	126.993	161 × 10^2^
Linear	Log_10_ADH	Log_10_ (Y) = 2.469 + 0.053x	0.744	0.141	0.020
Quadratic	Log_10_ADH	Log_10_ (Y) = 2.228 + 0.146x − 0.006x^2^	0.892	0.091	0.008
Logarithm	Log_10_ADH	Log_10_ (Y) = 2.295+ 0.327 Log_10_ (x)	0.911	0.083	0.007

SE: standard error of estimation, MSE: mean square error.

## Data Availability

Data is contained within the article or Supplementary Material.

## Supplementary Materials

The following are available online at https://www.mdpi.com/article/10.3390/insects12040323/s1, Table S1. Body lengths (mean ± SD, mm) for each stage of Lucilia sericata. Table S2. Minimum developmental times (mean ± SD, hours) for each stage of *Lucilia sericata*. Table S3. Minimum ADH values (mean ± SE, ADH) for each stage of *Lucilia sericata*.

## References

[1] Amendt J., Campobasso C.P., Goff M.L., Grassberger M. (2010). Current Concepts in Forensic Entomology.

[2] Byrd H., Castner J.L. (2010). Forensic Entomology: The Utility of Arthropods in Legal Investigations.

[3] Shin S.E., Jang M.S., Park J.H., Park S.H. (2015). A forensic entomology case estimating the minimum postmortem interval using the distribution of fly pupae in fallow ground and maggots with freezing injury. Korean J. Leg. Med..

[4] Davidson J. (1944). On the relationship between temperature and rate of development of insects at constant temperatures. J. Anim. Ecol..

[5] Gennard D.E. (2012). Forensic Entomology: An Introduction.

[6] Amendt J., Campobasso C.P., Gaudry E., Reiter C., LeBlanc H.N., Hall M.J. (2007). Best practice in forensic entomology—Standards and guidelines. Int. J. Legal Med..

[7] Tarone A.M., Foran D.R. (2008). Generalized additive models and *Lucilia sericata* growth: Assessing confidence intervals and error rates in forensic entomology. J. Forensic Sci..

[8] Smith K.G.V. (1986). A Manual of Forensic Entomology.

[9] Benecke M., Josephi E., Zweihoff R. (2004). Neglect of the elderly: Forensic entomology cases and considerations. Forensic Sci. Int..

[10] Arnaldos M.I., García M.D., Romera E., Presa J.J., Luna A. (2005). Estimation of postmortem interval in real cases based on experimentally obtained entomological evidence. Forensic Sci. Int..

[11] Saigusa K., Takamiya M., Aoki Y. (2005). Species identificaion of the forensically important flies in Iwate prefecture, Japan based on mitochondrial cytochrome oxidase gene subunit I (CO I) sequences. Legal Med..

[12] Bugelli V., Forni D., Bassi L.A., Paolo M.D., Marra D., Lenzi S., Toni C., Giusiani M., Domenici R., Gherardi M. (2015). Forensic entomology and the estimation of the minimum time since death in indoor cases. J. Forensic Sci..

[13] Shin S.E., Lee H.J., Park J.H., Ko K.S., Kim Y.H., Kim K.R., Park S.H. (2015). The first survey of forensically important entomofauna collected from medicolegal autopsies in South Korea. Biomed. Res. Int..

[14] Choe S., Lee D., Park H., Jeon H.K., Kim H., Kang J.H., Jee C.H., Eom K.S. (2016). Canine wound myiasis caused by *Lucilia sericata* (Diptera: Calliphoridae) in Korea. Korean J. Parasitol..

[15] Kamal A.S. (1958). Comparative study of thirteen species of sarcosaprophagous Calliphoridae and Sarcophagidae (Diptera) I. Bionomics. Ann. Entomol. Soc. Am..

[16] Ash N., Greenberg B. (1975). Developmental temperature responses of the sibling species *Phaenicia sericata* and *Phaenicia pallescens*. Ann. Entomol. Soc. Am..

[17] Greenberg B. (1991). Flies as forensic indicators. J. Med. Entomol..

[18] Anderson G.S. (2000). Minimum and maximum development rates of some forensically important Calliphoridae (Diptera). J. Forensic Sci..

[19] Grassberger M., Reiter C. (2001). Effect of temperature on *Lucilia sericata* (Diptera: Calliphoridae) development with special reference to the isomegalen- and isomorphen-diagram. Forensic Sci. Int..

[20] Marchenko M.I. (2001). Medicolegal relevance of cadaver entomofauna for the determination of the time of death. Forensic Sci. Int..

[21] Roe A., Higley L.G. (2015). Development modeling of *Lucilia sericata* (Diptera: Calliphoridae). PeerJ.

[22] VanLaerhoven S.L. (2008). Blind validation of postmortem interval estimates using developmental rates of blow flies. Forensic Sci. Int..

[23] Núñez-Vázquez C., Tomberlin J.K., Cantú-Sifuentes M., García-Martínez O. (2013). Laboratory development and field validation of *Phormia regina* (Diptera: Calliphoridae). J. Med. Entomol..

[24] Kanō R., Shinonaga S. (1968). Calliphoridae (Insecta: Diptera), Fauna Japonica.

[25] Tachibana S., Numata H. (2004). Effects of temperature and photoperiod on the termination of larval diapause in *Lucilia sericata* (Diptera: Calliphoridae). Zoolog. Sci..

[26] Rivers D.B., Thompson C., Brogan R. (2011). Physiological trade-offs of forming maggot masses by necrophagous flies on vertebrate carrion. Bull. Entomol. Res..

[27] Tomberlin J.K., Benbow M.E. (2015). Forensic Entomology: International Dimensions and Frontiers.

[28] Adams Z.J., Hall M.J. (2003). Methods used for the killing and preservation of blowfly larvae, and their effect on post-mortem larval length. Forensic Sci. Int..

[29] SAS Institute (2002). PROC User’s Manual, Version 9.1.

[30] Motulsky H., Christopoulos A. (2004). Fitting Models to Biological Data Using Linear and Nonlinear Regression: A Practical Guide to Curve Fitting.

[31] Richards C.S., Rowlinson C.C., Cuttiford L., Grimsley R., Hall M.J.R. (2013). Decomposed liver has a significantly adverse affect on the development rate of the blowfly *Calliphora vicina*. Int. J. Legal Med..

[32] Moreau G., Michaud J.P., Schoenly K.G., Tomberlin J.K., Benbow M.E. (2015). Experimental Design, Inferential Statistics, and Computer Modeling. Forensic Entomology: International Dimensions and Frontiers.

[33] Johnson A.P., Wallman J.F. (2014). Effect of massing on larval growth rate. Forensic Sci. Int..

[34] Wells J.D., Lecheta M.C., Moura M.O., LaMotte L.R. (2015). An evaluation of sampling methods used to produce insect growth models for postmortem interval estimation. Int. J. Legal Med..

[35] LaMotte L.R., Roe A.L., Wells J.D., Higley L.G. (2017). A statistical method to construct confidence sets on carrion insect age from development stage. J. Agric. Biol. Environ. Stat..

[36] Goodbrod J.R., Goff M.L. (1990). Effects of larval population density on rates of development and interactions between two species of *Chrysomya* (Diptera: Calliphoridae) in laboratory culture. J. Med. Entomol..

[37] Charabidze D., Bourel B., Gosset D. (2011). Larval-mass effect: Characterisation of heat emission by necrophageous blowflies (Diptera: *Calliphoridae*) larval aggregates. Forensic Sci. Int..

[38] Kotzé Z., Villet M.H., Weldon C.W. (2015). Effect of temperature on development of the blowfly, *Lucilia cuprina* (Wiedemann) (Diptera: *Calliphoridae*). Int. J. Legal Med..

[39] Arnold C.Y. (1959). The determination and significance of the base temperature in a linear heat unit system. Proc. Am. Soc. Hortic. Sci..

[40] Reibe S., Doetinchem P.V., Madea B. (2010). A new simulation-based model for calculating post-mortem intervals using developmental data for *Lucilia sericata* (Dipt.: *Calliphoridae*). Parasitol. Res..

[41] Marchenko M.I. (1985). Development of *Chrysomyia albiceps* WD. (Diptera, *Calliphoridae*). Entomol. Rev..

[42] Greenberg B., Kunich J.C. (2002). Entomology and the Law: Flies as Forensic Indicators.

[43] Tarone A.M., Foran D.R. (2011). Gene expression during blow fly development: Improving the precision of age estimates in forensic entomology. J. Forensic Sci..

[44] Park J.E., Shin S.E., Park S.H., Jeong S.J., Park S.H., Moon T.Y., Lee J.W. (2019). A study for estimating growth time of *Calliphoridae* flies using statistical models. J. Sci. Crim. Investig..

