# Supplementary material for: The Growth Model of Forensically Important Lucilia sericata (Meigen) (Diptera: Calliphoridae) in South Korea
Source: Insects. 2021 Apr 6;12(4):323. doi: 10.3390/insects12040323 (PMC8067387; doi:10.3390/insects12040323)
Supplement: Supplementary file 1 [file insects-12-00323-s001.pdf]

Table S1. Body lengths (mean ± SD, mm) for each stage of *Lucilia sericata*.

| Stage | 20°C | 22°C | 24°C | 26°C | 28°C | 32°C | 35°C |
|---|---|---|---|---|---|---|---|
| Egg | 1.22±0.09 | 1.06±0.16 | 1.23±0.11 | 1.08±0.10 | 1.21±0.12 | 1.22±0.12 | 1.20±0.06 |
| 1st instar | 2.55±0.55 | 2.29±0.62 | 2.76±0.57 | 2.16±0.71 | 2.50±0.73 | 2.45±0.75 | 2.45±0.80 |
| 2nd instar | 6.43±1.59 | 6.10±1.31 | 6.85±1.67 | 6.04±1.39 | 6.38±1.42 | 6.02±1.65 | 6.05±1.57 |
| 3rd instar | 13.79±2.08 | 12.27±2.36 | 14.06±1.85 | 11.60±2.56 | 13.41±2.15 | 13.45±2.31 | 12.79±2.80 |
| Post-feeding | 12.84±1.80 | 11.89±2.80 | 12.98±2.06 | 11.20±2.18 | 11.93±1.78 | 12.41±1.67 | 11.90±1.67 |
| Pupa | 8.11±0.42 | 7.46±1.35 | 7.84±0.51 | 7.45±0.93 | 7.75±0.45 | 7.76±0.43 | 7.42±0.64 |

Table S2. Minimum developmental times (mean ± SD, hours) for each stage of *Lucilia sericata*.

| Stage | 20°C | 22°C | 24°C | 26°C | 28°C | 32°C | 35°C |
|---|---|---|---|---|---|---|---|
| Egg | 28.8 ± 6.6 | 24.0 ± 0.0 | 24.0 ± 0.0 | 19.2 ± 6.6 | 14.4 ± 5.4 | 12.0 ± 0.0 | 12.0 ± 0.0 |
| 1st instar | 33.6 ± 10.0 | 24.0 ± 0.0 | 21.6 ± 10.0 | 16.8 ± 6.6 | 21.6 ± 5.4 | 14.4 ± 5.4 | 12.0 ± 0.0 |
| 2nd instar | 40.8 ± 6.6 | 26.4 ± 5.4 | 31.2 ± 10.7 | 21.6 ± 5.4 | 24.0 ± 8.5 | 21.6 ± 5.4 | 19.2 ± 6.6 |
| 3rd instar | 79.2 ± 18.2 | 45.6 ± 5.4 | 48.0 ± 14.7 | 38.4 ± 5.4 | 43.2 ± 6.6 | 36.0 ± 0.0 | 43.2 ± 6.6 |
| Post-feeding | 81.6 ± 13.1 | 87.0 ± 6.0 | 48.0 ± 19.0 | 78.0 ± 15.5 | 48.0 ± 12.0 | 69.6 ± 19.7 | 69.6 ± 15.6 |
| Pupa | 230.5 ± 49.9 | 187.0 ± 37.0 | 181.8 ± 13.3 | 132.0 ± 20.3 | 129.6 ± 26.0 | 108.0 ± 19.0 | 100.8 ± 20.1 |
| Egg to Adult | 494.5 ± 36.7 | 394.0 ± 37.0 | 354.6 ± 14.6 | 306.0 ± 23.0 | 280.8 ± 20.2 | 261.6 ± 13.2 | 256.8 ± 10.8 |

Table S3. Minimum ADH values (mean ± SE, ADH) for each stage of *Lucilia sericata*.

| Stage | 20°C | 22°C | 24°C | 26°C | 28°C | 32°C | 35°C |
|---|---|---|---|---|---|---|---|
| E to 1L | 316.8±32.3 | 312.0±0.0 | 360±0.0 | 326.4±44.3 | 273.6±45.6 | 276.0±0.0 | 312.0±0.0 |
| 1L to 2L | 369.6±49.4 | 312.0±0.0 | 324.0±67.4 | 285.6±44.3 | 410.4±45.6 | 331.2±55.2 | 312.0±0.0 |
| 2L to 3L | 448.8±32.3 | 343.2±26.6 | 468.0±72.0 | 367.2±36.2 | 456.0±72.1 | 496.8±55.2 | 499.2±76.4 |
| 3L to P3L | 871.2±89.5 | 592.8±26.6 | 720.0±98.6 | 652.8±36.2 | 820.8±55.9 | 828.0±0.0 | 1,123.2±76.4 |
| P3L to P | 897.6±64.7 | 1,131.0±33.0 | 720.0±127.3 | 1,326.0±116.3 | 912.0±102.0 | 1,600.8±202.8 | 1,809.6±181.9 |
| P to A | 2,535.5±245.4 | 2,431.0±203.5 | 2,727.0±89.3 | 2,244.0±152.3 | 2,462.4±221.1 | 2,484.0±195.2 | 2,620.8±233.5 |

E: egg; 1L: 1st instar; 2L: 2nd instar; 3L: 3rd instar; P3L: Postfeeding 3rd instar; P: Pupa; A: Adult
